# Challenging Density Functional Theory Calculations with Hemes and Porphyrins

**DOI:** 10.3390/ijms17040519

**Published:** 2016-04-07

**Authors:** Sam P. de Visser, Martin J. Stillman

**Affiliations:** 1 Manchester Institute of Biotechnology and School of Chemical Engineering and Analytical Science, the University of Manchester, 131 Princess Street, Manchester M1 7DN, UK; 2 Department of Chemistry, The University of Western Ontario, London, ON N6A 5B7, Canada

**Keywords:** DFT, enzyme mechanism, MCD spectroscopy, Ga(III)PPIX, chlorophylls

## Abstract

In this paper we review recent advances in computational chemistry and specifically focus on the chemical description of heme proteins and synthetic porphyrins that act as both mimics of natural processes and technological uses. These are challenging biochemical systems involved in electron transfer as well as biocatalysis processes. In recent years computational tools have improved considerably and now can reproduce experimental spectroscopic and reactivity studies within a reasonable error margin (several kcal·mol^−1^). This paper gives recent examples from our groups, where we investigated heme and synthetic metal-porphyrin systems. The four case studies highlight how computational modelling can correctly reproduce experimental product distributions, predicted reactivity trends and guide interpretation of electronic structures of complex systems. The case studies focus on the calculations of a variety of spectroscopic features of porphyrins and show how computational modelling gives important insight that explains the experimental spectra and can lead to the design of porphyrins with tuned properties.

## 1. Introduction

In this paper we will provide a brief overview concerning the application of computational methods to complex experimental properties of tetrapyrroles with the aim of proving an understanding and even predictions of the chemically important features. We have focused on two closely related areas of research: first, the subtle reactivity-tuning of heme enzymes to enable control of the natural processes and also of disease states; and second, computationally-based guides to the design of synthetic porphyrinoid compounds with specific optical and redox properties for technological use. Thus, the porphyrin rings in heme enzymes and heme proteins are found with many different structures and orientations, which affect their spectroscopic as well as redox properties. The powerful combination of the superior assignment criteria of experimental magnetic circular dichroism (MCD) spectral data with time-dependent density functional theory (TDDFT) results has led to the development relationships that can predict optical and redox properties with greater reliability than previously possible. Understanding spectroscopic and structural differences is important as hemes have not only the well-known and vitally important functions in nature, but as well as in Biotechnology and Engineering, for instance in fuel cells, electron transfer systems and in catalysis.

In the following, we touch on just four examples to showcase the major progress that has been made in strength and stability of computational modelling of the spectroscopic features of porphyrins and hemes, which is leading to important contributions in the field and assists in experimental data interpretation and in the structural design of future tetrapyrroles.

Introduction to Cases 1 and 2: heme enzymes and reactivity. Heme enzymes have important functions in biosystems ranging from oxygen transport (as in hemoglobin), electron transfer (e.g., cytochrome *c*), the detoxification of hydrogen peroxide (for example peroxidases) as well as substrate monoxygenation [1,2,3,4,5,6]. A large class of enzymes involved in the latter type of reactions is the cytochromes P450, which are highly versatile and catalyze a range of oxygen atom transfer reactions to substrates in the body as a means to initiate their metabolism in the body [7,8,9,10,11,12]. As such they function in the human liver to initiate the catabolism of drug molecules and hence are well studied by the pharmaceutical industry [13,14]. In addition, P450 enzymes are involved in biosynthetic reaction pathways of hormones, such as estrogen, in the liver [15,16]. The reactions catalyzed by P450 isozymes in the body include the hydroxylation of aliphatic groups, the epoxidation of C=C double bonds, the sulfoxidation of sulfides and the hydroxylation of arenes [17,18,19]. Their structure and reactivity is still poorly understood and questions that, in particular, are being investigated in chemical biology, biological chemistry and bioinorganic chemistry relate to how structural differences lead to functional changes through seemingly similar cofactor architecture.

**Figure 1:**
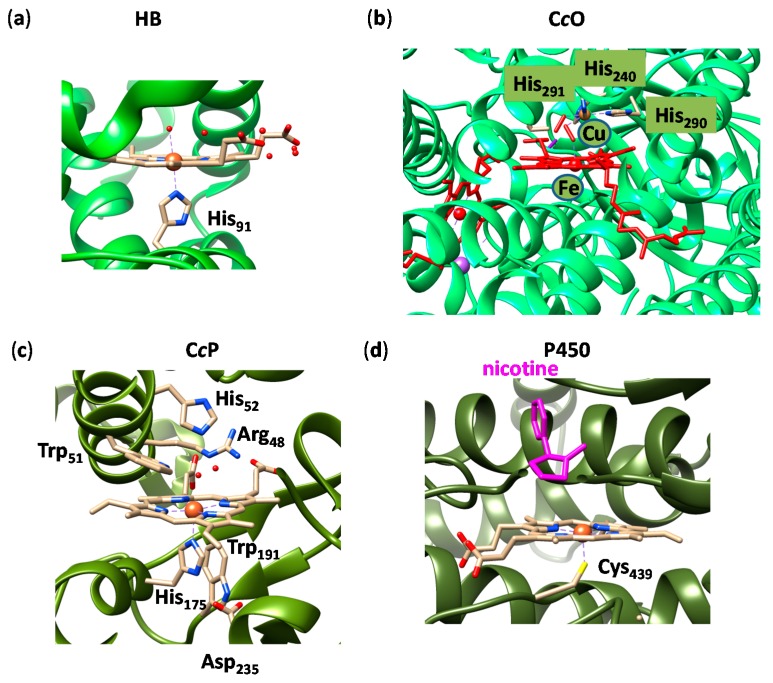
Active site structures of typical iron-heme enzymes. (**a**) hemoglobin (HB), 2QSP pdb (protein databank); (**b**) cytochrome *c* oxidase (C*c*O), 3WG7 pdb; (**c**) cytochrome *c* peroxidase (C*c*P), 4A6Z pdb; (**d**) cytochrome P450 with nicotine bound, 4EJG pdb. Colored ribbons represent the protein backbone. Key residues and substrate (nicotine) highlighted.

To highlight the dramatic difference in structure and geometry of various heme-containing enzymes, we display a selection of four common heme enzymes in Figure 1 as an example. The active site structures in Figure 1 are those of hemoglobin (a); cytochrome *c* oxidase (b); cytochrome *c* peroxidase (c) and cytochrome P450 (d) as taken from the 2QSP [20], 3WG7 [21], 4A6Z [22] and 4EJG [23] protein databank (pdb) files, respectively. These enzymes have dramatically different functions in biology, whereby hemoglobin transports O_2_ molecules through the blood stream from the lungs to the muscles and organs [24]. The heme group in hemoglobin is, therefore, located on the surface of the protein and molecular oxygen will bind the sixth metal ligand position trans to His_91_. In cytochrome *c* peroxidase (C*c*P), by contrast, the heme is buried inside the protein and is involved in the detoxification of hydrogen peroxide, which in a catalytic cycle is first converted into an iron(IV)-oxo heme cation radical called Compound I (CpdI) and subsequently further reduced to another water molecule [25,26]. The axial ligand trans to the hydrogen peroxide binding position to the metal is still a histidine group (His_175_ in the 4A6Z pdb file), but it is part of a hydrogen bonding triad that includes the carboxylate group of Asp_235_ and the NH proton of the indole ring of Trp_191_. In particular, the Trp_191_ residue in the active site of C*c*P is expected to influence the electronic properties of the heme group. Thus, electron paramagnetic resonance (EPR) studies on CpdI of C*c*P showed it to have an electronic configuration representing an [Fe^IV^(O)(heme)His---Trp^+•^], whereas the enzyme ascorbate peroxidase with a virtually identical heme binding site had an electronic configuration of [Fe^IV^(O)(heme^+•^)His---Trp] instead [27,28]. Computational modelling showed this to originate from a nearby bound cation (at a distance of about 12 Å from the heme iron) in ascorbate peroxidase, which is absent in C*c*P [29]. Due to an induced electric field effect from the cation, *i.e.*, sodium, the electronic configuration of CpdI changed from a tryptophan radical (as in C*c*P) to a heme radical in ascorbate peroxidase. Note that the Arg_48_, His_52_ and Trp_51_ residues shown in Figure 1c are involved in hydrogen peroxide binding through a network of hydrogen bonding interactions and assist with its protonation and catabolic mechanism.

Also shown in Figure 1 is the active site structure of cytochrome *c* oxidase (C*c*O) (Part b), which is a membrane-bound protein involved in cellular respiration that catalyzes the reduction of O_2_ to water [30,31,32]. It has several heme groups as identified in red in the Figure. One of the hemes has a nearby nonheme copper center that is linked to the protein through interactions with three histidine residues: His_240_, His_290_ and His_291_. A second heme group nearby this nonheme copper center participates in the electron transfer mechanism and brings electrons into the active site of the iron-heme---nonheme copper center.

The final structure in Part d of Figure 1 is the active site of the substrate-bound (nicotine) iron(III)-heme complex of cytochrome P450, where the heme binds the protein via a cysteinate residue (Cys_439_). Note that in some P450 isozymes and structures the heme is close to planarity, whereas in others it is ruffled, saddled or domed [33,34]. Moreover, as can be seen from Figure 1, the propionate groups are pointing up (but are twisted) in HB and C*c*P, whereas they point down in the P450 structure. Whether the position of the propionate groups is important for function and catalysis is currently unknown. However, the nature of substituents bound to the heme will affect its electronic configuration, but also the spectroscopic properties. A natural result of this, of course, is that hemes have many differences in color in nature [35]. Despite the fact that all four structures in Figure 1 have a central heme group (or protoporphyrin IX) bound by an iron(III) ion, actually there are dramatic differences in geometric features. First of all, in some cases the fifth ligand is an imidazole group of a histidine residue, e.g., hemoglobin and peroxidases, whereas in the P450 structure its position is occupied by a cysteinate ligand [36].

Note that in catalases the heme group is bound to the protein via a tyrosinate linkage to the heme [37], and as such there is a large versatility of binding patterns associated with hemes. It has been proposed that the axial ligand incurs either a push-effect or a pull-effect of electron density [38] and, thereby affects the electron affinity of the oxidant. Indeed, computational modelling on the iron(IV)-oxo heme cation radical species, *i.e.*, Compound I (CpdI), of cytochrome *c* peroxidase, catalase and cytochrome P450 showed a dramatic drop in electron affinity (EA) of P450 CpdI as compared to the analogous peroxidase complex [39]. This was shown to affect the ability of the CpdI species of P450, cytochrome *c* peroxidase and catalase to abstract hydrogen atoms from a substrate and was proposed to be the reason only P450s are involved in substrate activation processes [40]. To take one step further, as shown by biomimetic CpdI model complexes, not only the reactivity is affected by the axial ligand description of the iron(IV)-oxo porphyrin cation radical, but also the spectroscopic parameters of the complex [41,42,43]. In particular, Green showed a linear correlation between the metal-oxo stretch vibration and its bond length [44].

**Figure 2:**
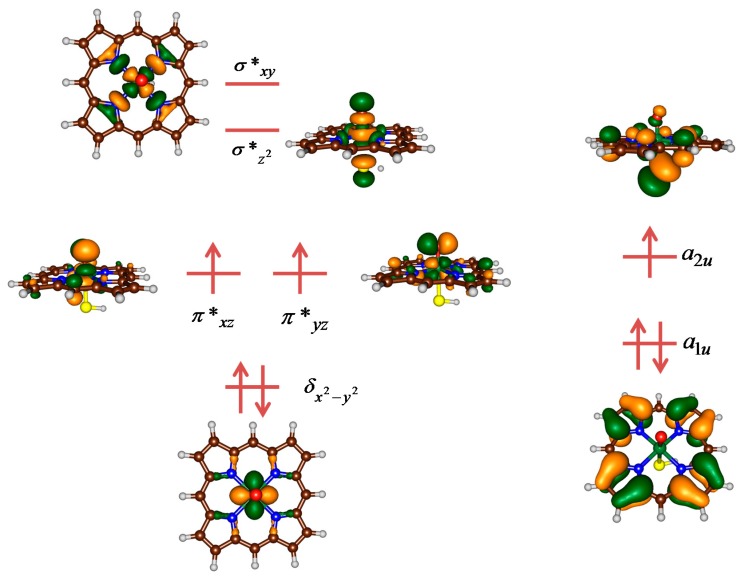
Occupied and virtual valence orbitals of P450 compound I (CpdI) in the quartet spin state. Molecular orbitals give positive and negative phases in green and gold.

One particular issue that makes the interpretation of heme and synthetic porphyrins difficult is the fact that these complexes can appear in various close lying spin states. Thus, P450 CpdI has a set of valence orbitals that are close in energy and lead to several low lying electronic and spin states. Figure 2 gives the metal-type and valence porphyrin-type molecular orbitals of [Fe^IV^(O)(Por^+•^)SH], which is used as a model for P450 CpdI with the heme replaced by a protoporphyrin IX (Por) without side chains [45]. On the left-hand-side we give the metal-type orbitals that are labelled according to the involvement of the 3d orbital on iron, whereas on the right-hand-side two nonbonding porphyrin orbitals are given. The metal-based orbitals from bottom to top are the nonbonding δ*_x2–y2_* orbital, the π* antibonding interactions between the metal and the 2p on oxygen (π*_xz_ and π*_yz_), the σ* antibonding interaction along the metal-oxo bond (σ*_z2_) and finally the σ* antibonding interactions of the metal with the porphyrin (σ*_xy_). Two nonbonding porphyrin orbitals complete the set of valence orbitals, namely a*_1u_* and a*_2u_*, where we use their symmetry labels under D*_4h_* symmetry. The full set of orbitals in Figure 2 is occupied with seven electrons, which generally gives a ^4,2^A_2u_ ground state with orbital occupation δ*_x2–y2_*^2^ π*_xz_^1^ π*_yz_^1^ a*_1u_*^2^ a*_2u_*^1^. However, the spin-orbit coupling between the π* and a*_2u_* orbitals is small and, therefore, the three unpaired electrons are either ferromagnetically coupled into an overall quartet spin state or antiferromagnetically coupled into an overall doublet spin state [46].

The fact that the ground state of P450 CpdI is a virtually degenerate doublet and quartet spin state, implies reactivity patterns on two spin state surfaces, and hence is labelled two-state-reactivity or more generally multistate-reactivity [47]. Therefore, a reaction mechanism of CpdI with a substrate will take place on competing spin state surfaces, each with their own mechanism and their own barrier heights (and rate constants) and consequently product distributions. In the past, we encountered examples where reaction mechanisms leading to by-products were possible on the quartet spin state but not on the doublet spin state [48,49]. As such, the two-state-reactivity can lead to product distributions that are different on each of the individual spin states. Obviously, the nature of the spin state affects reactivity patterns, but also spectroscopic variables. The local environment, such as hydrogen bonding interactions, for instance can affect the structure and consequently the properties of the heme group. As an example, recent work on synthetic iron(III)-chloride porphyrin complexes showed that hydrogen bonding donation affected the spin state splitting and reversed a spin state ordering [50]. The structure and reactivity is still poorly understood; however, in the absence of iron natural porphyrins photochemical reactions become important, for example, in chlorophyll. From a biotechnological point of view, mimicking the photochemical properties of the natural porphyrins offers a route to both singlet oxygen production, such as photodynamic therapy, and solar cells manufacture.

**Figure 3:**
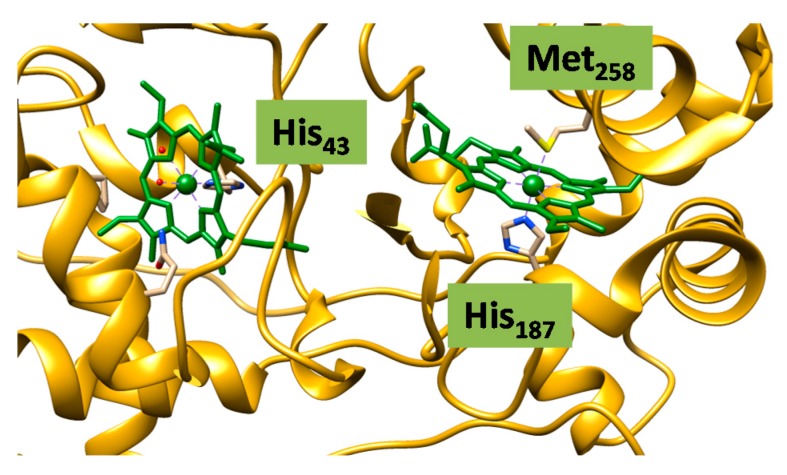
Extract of the di-heme cytochrome c peroxidase structure as taken from the 1IQC pdb file.

A final example on heme structures is shown in Figure 3, where we depict the active site structure of the di-heme cytochrome *c* peroxidase as taken from the 1IQC pdb file [51]. Thus, one of the hemes provides electrons to the catalytic heme, where the peroxidase reaction takes place. The electron transfer heme, right-hand-side of Figure 3 is ligated by a histidine (His_187_) and a methionine (Met_258_) residue and relays electrons to the other heme. Interestingly, the catalytic heme has a histidine axial ligand (His_43_) as expected but the above mentioned triad of a neighboring carboxylate and tryptophan residues as seen in the mono-heme structure in Figure 1c are missing. As such, the CpdI species is likely to be in a [Fe^IV^(O)(heme^+•^)His] configuration rather than a close-shell heme coupled to a tryptophan radical.

Introduction to Cases 3 and 4: Synthetic porphyrins. The remarkable properties of the natural iron porphyrins (heme b, heme c and many others) found throughout nature, the impact on all life on the planet of the chlorophylls and the highly specialized natural porphyrinoids, for example the enzymatic control afforded by the redox properties of the cobalt in the cobalamins, have been studied by many different techniques to both understand their reactivity and to learn how to mimic or duplicate these properties [52]. Even today novel natural porphyrinoids are being discovered for which the differences in reactivity needs understanding, for example chlorophyll *f* from cyanobacteria exhibits the lowest known Q band energy absorption band lying to the red of known chlorophylls. How do the different peripheral substituents make that change? This question has led to the extensive science of synthesizing novel porphyrinoids [53]. The phthalocyanines, tetraazatetrabenzporphyrins, were formally discovered and their characterization started in 1927 at Scottish Dyes Ltd. Their extensive and novel (at that time) properties were explored and reported on over many years by Linstead and coworkers at Imperial College, London [54]. The vitally important property of the color of tetrapyrroles, exemplified by the chlorophylls, and the technological uses of the phthalocyanines, also is the focus in the synthesis of novel compounds that can be used technologically, for example as solar cells [55] or for photodynamic therapy [56]. However, despite the research goal being for specific optical properties, the information obtained from absorption and emission spectral data is not often very helpful in determining the assignment of the spectrum of these large multifunctional molecules to specific states or the electronic structure. This means that the origins of the optical data observed are hard to pinpoint to a specific molecular component. For example, the optical spectrum of the chlorophylls still attracts study [57,58]. Magnetic circular dichroism spectroscopy has for over 50 years provided additional and very specific assignment criteria that do allow the optical data both absorption and emission to be interrogated and improved assignments to be obtained [59]. The coupling of those data with the electron density data and electron density distributions allows the functional effects of peripheral and ring modifications to be examined closely. Sometimes the TDDFT results do reliably account for the optical properties, but in many examples, those data are not good matches and the TDDFT results must be combined with assignments from other techniques. Cases 3 and 4 in this paper provide examples of the use of the optical data, electron density distributions of the top filled and lowest virtual molecular orbitals, together with TDDFT results to characterize the electronic structures of porphyrins of potential technological or therapeutic use.

## 2. Results

### 2.1. Case Study 1

To test the quality and reproducibility of density functional methods (DFT) in reproducing experimental rate constants, and consequently activation energies, of a chemical reaction, a combined low-pressure mass spectrometry and DFT study was performed on olefin epoxidation by [Fe^IV^(O)(Por^+•^)]^+^ (**1**) and [Fe^IV^(O)(TPFPP^+•^)]^+^ (**2**), whereby Por = porphyrin and TPFPP = tetrapentafluorophenylporphyrin [60]. Thus, complex **2** was synthesized in methanol/dichloromethane solution from the reaction of the iron(III) complex with iodosylbenzene. The solution was then inserted into a Fourier transform-ion cyclotron resonance (FT-ICR) mass spectrometer through electrospray ionization and mass selected in the ICR cell. Subsequently, neutral collision gases (olefins) were inserted into the mass spectrometer and the product abundances were monitored as a function of time, which enabled the determination of the first-order rate constants for the oxygen atom transfer reactions of 14 substrates.

**Figure 4:**
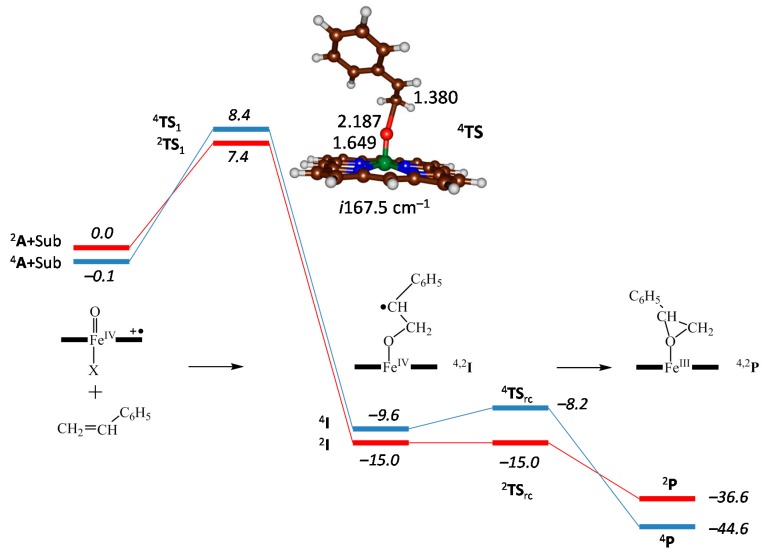
Potential energy landscape of styrene epoxidation by [Fe^IV^(O)(Por^+•^)Cl] as calculated with UB3LYP in Gaussian-09 with the quartet landscape in blue and the doublet landscape in red. Data taken from [61] and represents Δ*E* + ZPE values in kcal·mol^−1^ relative to isolated reactants. Optimized geometry of the **TS** gives bond lengths in angstroms.

Using DFT methods, thereafter, the reaction mechanism was modelled at the B3LYP level of theory and the enthalpy of activation for a set of oxygen atom transfer reactions was determined. As an example of a typical reaction profile and free energy landscape obtained for olefin epoxidation by **1** we display the calculated mechanism of styrene epoxidation by [Fe^IV^(O)(Por^+•^)Cl] in Figure 4 [61,62]. The reaction is stepwise with an initial C–O bond formation that gives a radical intermediate (**I**) via a transition state **TS1**. In a subsequent step the radical attacks the oxygen atom and closes the epoxide ring to form products **P** via a ring-closure transition state **TS**_rc_. The initial C–O bond formation barrier **TS1** is rate determining and the ring-closure barrier or very small or negligible. This is the same for a range of substrates and P450 CpdI mimics tested [60,61,62,63,64,65,66,67].

**Figure 5:**
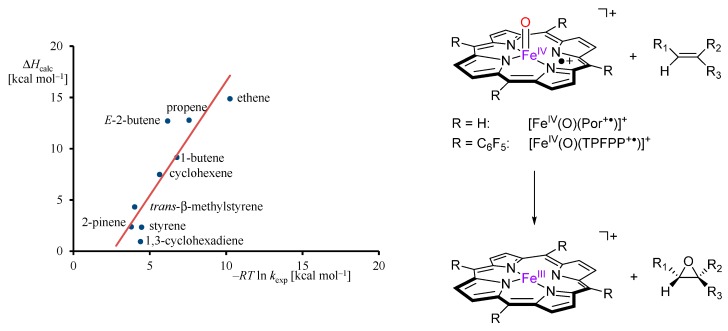
Correlation of experimental (FT-ICR MS) obtained rate constants *versus* computational barrier heights for olefin epoxidation by iron(IV)-oxo porphyrin cation radical models.

All substrates reacted efficiently through oxygen atom transfer to give the corresponding olefins. Figure 5 gives a plot of the correlation of the experimental and computational enthalpy of activation for olefin epoxidation by these iron(IV)-oxo porphyrin cation radical complexes [60]. As can be seen the trend is linear and most experimentally determined enthalpies of activation are reproduced within 1.5 kcal·mol^−1^ and a standard deviation of 3.4 kcal·mol^−1^. Therefore, DFT predicts experimentally determined reactivity trends well and similar ordering and reactivity patterns as those observed experimentally in the gas-phase under Ideal Gas conditions.

Technically, the reaction of cyclohexane or propene with iron(IV)-oxo porphyrin cation radical complexes can lead to a mixture of products originating from olefin epoxidation and aliphatic hydroxylation. Earlier DFT studies on the regioselectivity of aliphatic *versus* aromatic hydroxylation of propene by [Fe^IV^(O)(Por^+•^)X] with X = SH^−^ or Cl^−^ showed a dominant olefin epoxidation pathway by several kcal·mol^−1^ [64,65,66]. However, inclusion of solvent effects as well as hydrogen bonding interactions towards the thiolate group of the cysteinate axial ligand [68] actually stabilized the aliphatic hydroxylation pathways over the epoxidation pathways and led to a complete regioselectivity switch. Interestingly, the mass spectrometric results confirm dominant double bond epoxidation over hydroxylation, whereas in enzymatic systems typically P450 reacts to form alcohol products in a reaction with propene [69]. As such, it appears B3LYP calculations on the regioselectivity of aliphatic hydroxylation *versus* olefin epoxidation predict the correct regioselectivity preference with enthalpies of activation that match Ideal Gas determined values from low-pressure mass spectrometry very well.

Further benchmark studies on experimental *versus* computational determined reaction rates and free energies of activation were done on several nonheme iron(IV)-oxo systems [70,71,72,73]. In general, the correct product distributions are obtained but free energies of activation have a systematic error of about 3–5 kcal·mol^−1^. The key, however, is that the correct product distributions are predicted even in cases where multiple reaction products are possible. Indeed, this enabled us to predict the factors that determine the enantioselectivity of the enzyme *S*-mandalate synthase and bioengineered it to *R*-mandalate synthase through modelling predictions [74].

### 2.2. Case Study 2

As mentioned above high-valent metal-oxo species are common intermediates in enzymes and, in particular, CpdI of P450 is an important biochemical catalyst. A special ligand system that is highly suitable for stabilizing high-valent metal-oxo species with the metal in oxidation state IV or V are the corrole [75,76,77] and corrolazine [78] ligands. The corrole group has analogy to the porphyrin manifold, but lacks one of the meso-CH groups and, therefore, directly links two pyrrole units. The corrolazine, in addition to the missing meso-CH group has the other three meso-CH groups replaced by nitrogen atoms. As a consequence, corrole and corrolazine ligands have an overall charge of +3, whereas heme, protoporphyrin IX and phthalocyanine rings only have a charge of +2 [79].

Using a manganese corrolazine (Cz) system, a manganese(V)-oxo species was generated and spectroscopically characterized [80]. Subsequently, reactivity studies were performed with model substrates and, for instance, a dehydrogenation reaction was investigated with 9,10-dihydroanthracene and a sulfoxidation reaction of thioanisoles [80,81]. Interestingly, addition of anions, X^−^ with X = F^−^/CN^−^, led to a dramatic increase of the reaction rates, whereby the ratio of the second-order rate constants k_2_ gave a rate enhancement of 2100 when F^−^ was added to the system and 16,000 upon addition of CN^−^. To understand the factors that contribute to the rate enhancement, a density functional theory (DFT) study was performed [80].

**Figure 6:**
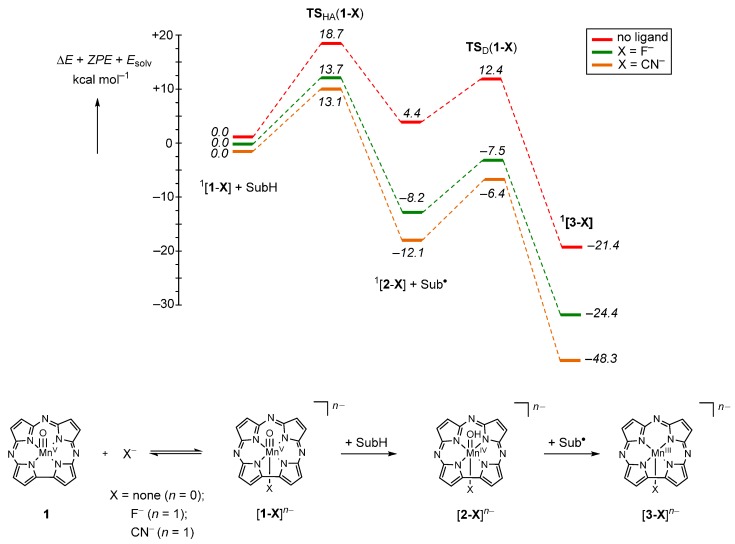
Catalytic mechanism of axial ligand binding and the subsequent dehydrogenation of substrate (9,10-dihydroanthracene) to water and anthracene. Energies are Δ*E* + ZPE values in kcal·mol^−1^ as calculated with B3LYP. Data taken from [80].

Figure 6 displays the overall reaction mechanism and the calculated potential energy profile of dehydrogenation of 9,10-dihydroanthracene (SubH) by [Mn^V^(O)(Cz)] (**1**) and [Mn^V^(O)(Cz)(X)]**^−^** ([**1^−^X**]**^−^**, X = F**^−^**/CN**^−^**). In all cases, the reaction is stepwise with an initial hydrogen atom abstraction barrier (**TS**_HA_) leading to a radical intermediate [**2–X**], which is followed by another hydrogen atom abstraction barrier (**TS**_D_) to form the manganese(III)-corrolazine complex [**3–X**] and anthracene products. The hydrogen atom abstraction barrier **TS**_HA_ is the rate determining step in the mechanism, while **TS**_D_ is much smaller. Indeed, experimental studies showed the reaction to proceed with a large kinetic isotope effect (KIE) of 10.4 and 6.7 for [**1–F**]^−^ and [**1–CN**]^−^, respectively. Calculations then estimated the KIEs from the reaction mechanism using the Eyring and Wigner models [82] and found values of 8.5 and 8.6, respectively, which is in good quantitative agreement with experiment.

All reactant structures are characterized, either with or without axial F^−^/CN^−^ ligand, as a closed-shell singlet spin state with orbital occupation δ*_x2_*_–*y2*_^2^ [80,81,83,84,85]. Higher in energy are a triplet spin manganese(V)-oxo species with δ*_x2_*_–*y2*_^1^ π*_xz_^1^ occupation and triplet and quintet spin states with δ*_x2_*_–*y2*_^1^ π*_xz_^1^ π*_yz_^1^ a”^1^ occupation. Addition of substituents to the periphery of the corrolazine macrocycle had little effect on the spin state ordering and relative energies [84] and neither had the addition of an axial ligand [80,84]. However, addition of Zn^2+^ ions to the solution led to the formation of a [Zn^2+^**–**O=Mn^IV^(Cz^+•^)] complex where the zinc pulled the oxygen atom away from the manganese corrolazine and forced it into a high-spin state with δ*_x2_*_–*y2*_^1^ π*_xz_^1^ π*_yz_^1^ a”^1^ occupation through valence tautomerism [83].

The hydrogen atom abstraction barriers from 9,10-dihydroanthracene are 18.7 kcal·mol^−1^ for structure **1**, 13.7 kcal·mol^−1^ for [**1–F**]^−^ and 13.1 kcal·mol^−1^ for [**1–CN**]^−^. Therefore, the rate enhancement for addition of an axial ligand to the manganese(V)-oxo corrolazine is 5.0 kcal·mol^−1^ for a fluoride anion and 5.6 kcal·mol^−1^ for a cyanide anion. By contrast, the experimental rate enhancements of the second-order rate constants of 2100 and 16,000 correspond to a decrease of the hydrogen atom abstraction barriers by 4.5 and 5.7 kcal·mol^−1^, respectively. Clearly, DFT computed reaction mechanisms and potential energy profiles reproduce relative energies of barrier heights and regioselectivities extremely well. However, as a caveat it should be mentioned that careful consideration of the methods and basis set needs to be taken into account [84,85,86,87].

### 2.3. Case Study 3

The challenges of computational analysis of heme proteins are compounded by the fact that the central iron atom can appear in a number of oxidation states (II, III or IV) as well as associated spin states as described above. Modelling heme-protein interactions in the absence of the iron(III) ion can in some cases lead to detailed information about the protein environment. However, great care should be taken in this approach as, for instance replacing the iron(III) with the commonly used zinc(II) does not provide a +3 charge of the metal-heme and therefore may not be particularly useful [59,88]. An alternative approach applied by us and others previously [89,90,91] is to replace iron(III) with gallium(III), which retains the correct charge. As such Ga(III)-protoporphyrin IX (Ga-PPIX) can function as a suitable model for ferric heme. Note that these and other gallium(III)-porphyrinoid complexes, such as gallium(III)-corroles, are used in medicine for tumor detection and elimination [92].

In addition, electronic coupling of the central iron in heme to other redox active metals may affect the spectroscopic fingerprints of the iron-heme. Thus, as described above in Figure 1b, the iron(III)-heme in cytochrome *c* oxidase has a close-lying nonheme copper(II) center. This is not an unusual feature as shown in Figure 3 for the di-heme structure of cytochrome *c* peroxidase, where the interactions between parallel heme rings leads to complicated electronic coupling [51,93]. In these heme enzymes the peroxidase activity is modulated by the electronic coupling between the two individual heme rings. Such interactions are very hard to analyze computationally. Models are first required to extend the analysis to the scale of two interacting porphyrin rings, and then the coupling of the redox active metals can be added. An approach to this is the use of Ga(III) in place of the Fe(III), clearly restricting interactions between the metal centers but allowing ring to ring effects to be examined [94]. Another example of the importance of heme-heme interactions is in the malarial hemozoin pigment that leads to insoluble ferric heme [95,96]. During its life cycle hemoglobin is degraded and the dimeric heme-based hemozoin is formed [97]. In a collaborative study, Bohle and Dodd prepared a Ga(III) substituted porphyrin and Pinter and Stillman carried out a spectroscopic and computational analysis [98]. The corresponding Zn(II)-PPIX was also prepared as a model for the spectroscopic and electronic structural properties of the ring with minimal interactions from the central metal. The absorption, and particularly, the magnetic circular dichroism (MCD) spectroscopic results showed that the electronic structures of the Ga(III)-PPIX and the Zn(II)-PPIX are similar but differ in terms of the absence of overlapping charge-transfer bands from the Fe(III)-PPIX, *i.e.*, heme. The Ga(III)-PPIX was inserted into apo-myoglobin and exhibited similar spectral data as compared to wildtype [91], which implicates that the Ga(III)-PPIX system is a suitable model for Fe(III) for testing porphyrin charge effects.

**Figure 7:**
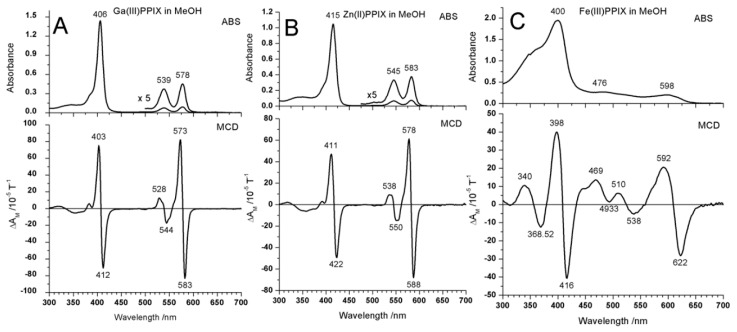
Absorption and magnetic circular dichroism (MCD) spectra of (**A**) Ga(III)-PPIX, (**B**) Zn(II)-PPIX and (**C**) Fe(III)-PPIX all in methanol. Reproduced with permission of the American Chemical Society from [84].

The absorption and MCD data measured in methanol for Ga(III)-PPIX, Zn(II)-PPIX and Fe(III)-PPIX are shown in Figure 7. It can be seen that the Ga(III)-PPIX and Zn(II)-PPIX display the same series of bands, namely Q bands at 578 and 583 nm, with the vibronic bands approximately 39 nm blue-shifted, and B bands at 406 and 415 nm, respectively. On the other hand, the Fe(III)-PPIX spectral data of the π-ring was overlaid by ring to Fe(III) charge-transfer, which is especially noticeable in the 476 nm region. The MCD spectral data identifies the *ca.* 9:1 ratio of angular momentum difference for the Q:B bands for the Ga(III) and Zn(II) complexes [99]. The effect of the charge-transfer is that it quenches the angular momentum through configuration interaction between the many available states in the visible region. The spectral data are consistent with a small but non-zero energy gap between the HOMO and HOMO − 1 orbitals, Δ{HOMO − (HOMO − 1)} [100]. In the following, we will quantify this statement using Figure 9 and Figure 11 that bring together 17 test systems for comparison.

**Figure 8:**
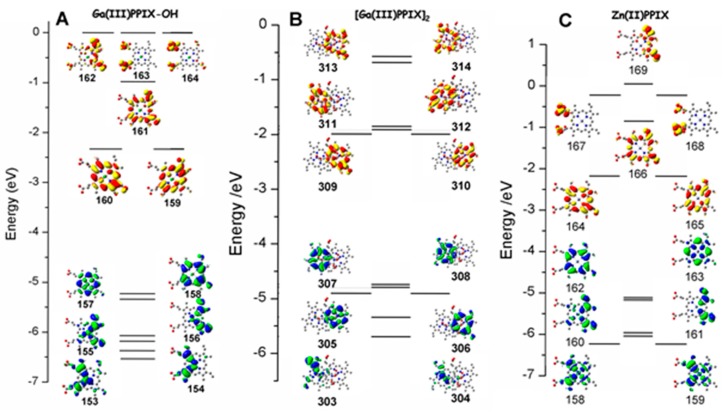
Top six highest occupied and lowest six unoccupied MOs for (**A**) Ga(III)-PPIX(OH) (orbitals 153–164), (**B**) the Ga(III)-PPIX dimer {(Ga(III)-PPIX)_2_} (orbitals 303–314) and (**C**) Zn(II)-PPIX (orbitals 158–169). The energies were calculated using density functional theory (DFT) methods: B3LYP/LANL2DZ. Shown are the energies and molecular orbital surfaces for the top six occupied MOs (green and blue phases) and the lowest six unoccupied MOs (red and yellow phases). Hydrogens have been omitted from structures for clarity. The lowest shown molecular orbital for Ga-PPIX is HOMO-5 at 153, for (Ga-PPIX)_2_ is HOMO-5 at 303, and for Zn-PPIX is HOMO-5 at 158. Reproduced with permission of the American Chemical Society [98].

The computational results on Ga(III)-PPIX, its dimer and Zn(II)-PPIX are shown in Figure 8. Although the PPIX ring is not centrosymmetric due to the peripheral substituents, there is little evidence in the optical data that they contribute significantly because the effect is observed to a greater extent below the top two occupied molecular orbitals (MOs), namely orbital 157 and 158 for Ga-PPIX and orbital 162 and 163 for Zn-PPIX. These highest occupied MOs (HOMOs) are almost identical in the electronic distribution to the MOs of the symmetric Zn-porphyrins [100,101]. The lower symmetry introduced by the peripheral substituents of PPIX also does not significantly affect the energies of the lowest unoccupied MOs (LUMOs), namely LUMO and LUMO + 1 orbitals. The MCD spectral data shows that those two orbitals are close enough to degeneracy to result in the appearance of the well-defined sigmoidal morphology of the symmetrical A term. The authors [98] reported that the influence of the asymmetric peripheral substituents typical of a PPIX ring, is seen at LUMO + 3 and higher for both Ga(III)-PPIX and Zn(II)-PPIX.

**Figure 9:**
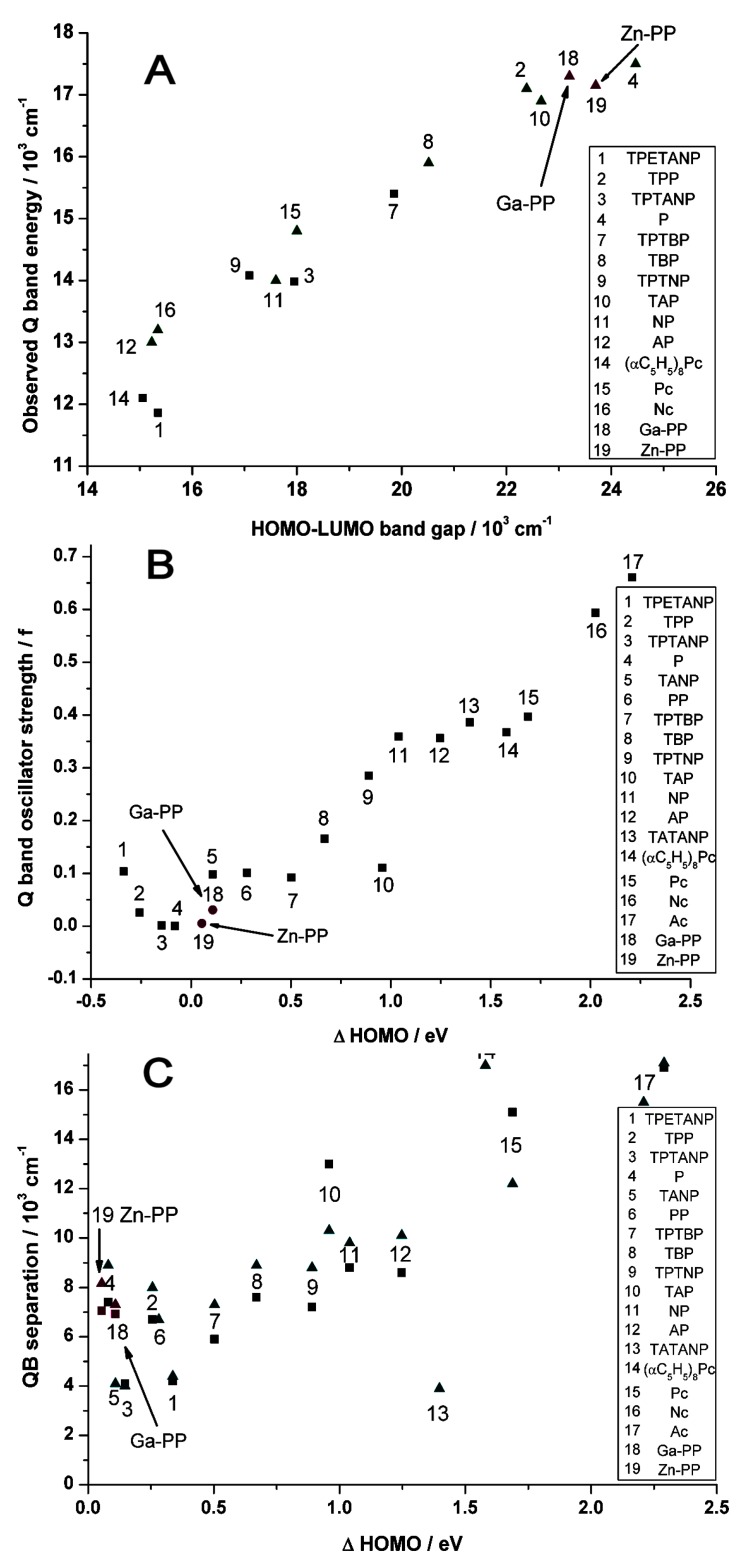
Trends in optical properties as a function of the energies of the four "Gouterman" orbitals. (**A**) Energy of the Q-band as a function of Δ(LUMO–HOMO); (**B**) Q-band oscillator strength (f) as a function of Δ(“HOMO”) or Δ{(HOMO) **−** (HOMO **−** 1)}; (**C**) Q-B energy gap as a function of Δ(“HOMO”) or Δ{(HOMO) **−** (HOMO **−** 1)}. (Key: Molecular structures of the zinc complexes of porphyrin (P), tetraazaporphyrin (TAP), tetraphenyl-porphyrin (TPP), tetrabenzoporphyrin (TBP), phthalocyanine (Pc), tetraphenyltetrabenzoporphyrin (TPTBP), naphthoporphyrin (NP), naphthalocyanine (Nc), tetraphenyltetranaphthoporphyrin (TPTNP), tetraacenaphthoporphyrin (TANP), tetraacenaphthotetraazaporphyrin (TATANP), tetraphenyltetraacenaphthoporphyrin (TPTANP), tetraphenylethynylacenaphthoporphyrin (TPETANP), tetraphenanthroporphyrin (PP), octa−henylphthalocyanine ((αC_5_H_5_)_8_Pc), anthracoporphyrin (AP) and anthracocyanine (Ac)). Adapted from [98].

Table 1 shows the calculated values for the energy splitting between the HOMO and (HOMO − 1) as well as the LUMO and (LUMO + 1) orbitals for the Ga(III) and Zn(II)-PPIX complexes. As has been reported previously [100], when the Δ(“HOMO”) value is close to zero, the oscillator strength of the Q-band transition is very low. The trend graph (Figure 9) places these data on the values for 17 porphyrins and phthalocyanines and the PPIX data follow the trend well. Similarly, the Δ{LUMO **−** HOMO} value predicts the Q-band energies closely near 17,000 cm^−1^ or 588 nm, almost exactly where the Q-bands are located by the MCD positive A-terms (Figure 7). The trend in the Q-B separation, while predicted accurately by the calculations as *ca*. 7000–8000 cm^−1^, however, do not lie on the trend line for the other test cases used.

**Figure 10:**
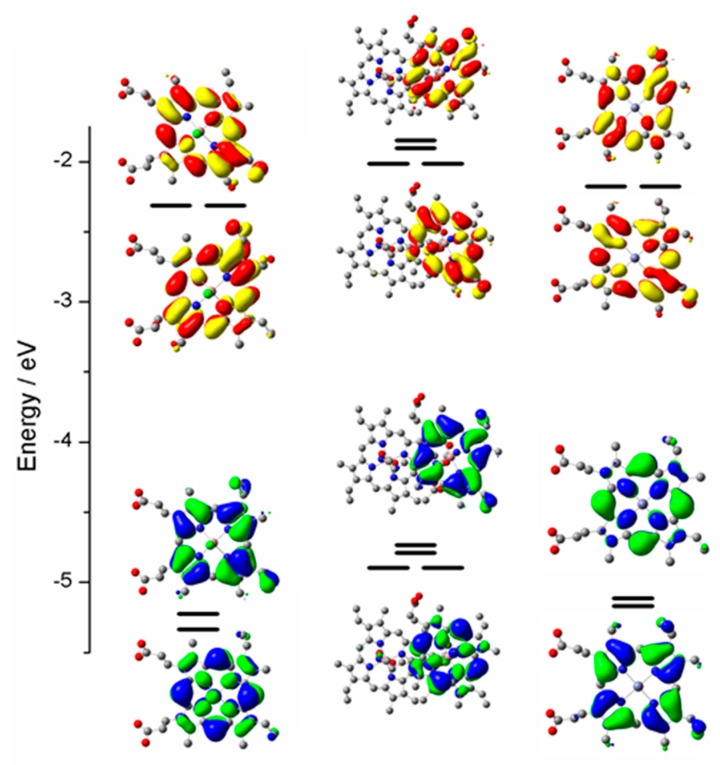
Relative energies of the four Gouterman orbitals of Ga(III)-PPIX, (Ga(III)-PPIX)_2_ and Zn(II)-PPIX. Only the electron density distribution of top ring of the dimer is shown for the (Ga(III)-PPIX)_2_. Colored lobes represent positive and negative phases of the orbitals. For the occupied molecular orbitals the two phases are indicated by green and blue isosurfaces; for the unoccupied molecular orbitals the two phases are indicated by red and yellow isosurfaces. Reproduced with permission of American Chemical Society [98].

In order to understand the trends depicted in Figure 9, it is instructive to view the four Gouterman orbitals in greater detail so as to be able to discern the nodal patterns that identify the HOMO and its partner with the ±4 and the LUMO and its partner with the ±5 origins angular momentum. These four Gouterman orbitals are schematically depicted in Figure 10. Indeed, locating the four nodes of the HOMO and the 5 nodes of the LUMO provide excellent evidence of the number of aromatic π-electrons, here confirming unambiguously that PPIX is an 18π-electron system. Figure 10 redraws the data in Figure 8 to allow the nodal patterns to be seen. The dimer nodes for the top ring only are shown. It is significant we feel that there was no significant delocalization over the two porphyrin rings, however, this is not unexpected of the dimer.

**Figure 11:**
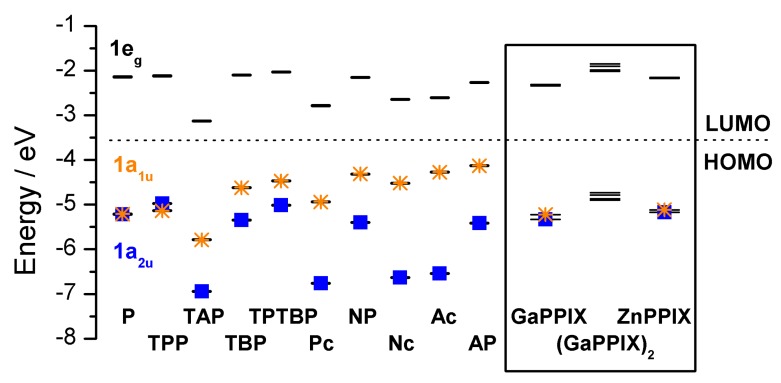
Comparison between the orbital energy levels (horizontal lines) of the “Gouterman” 4-orbitals for a range of porphyrin and phthalocyanines. The “*” marks the a_1u_ orbital. The key to the abbreviations is in the caption to Figure 9. The black box highlights the molecular orbitals energy levels of the GaPPIX, ZnPPIX, and the (GaPPIX)2. Reproduced with permission of American Chemical Society [100].

**Figure 12:**
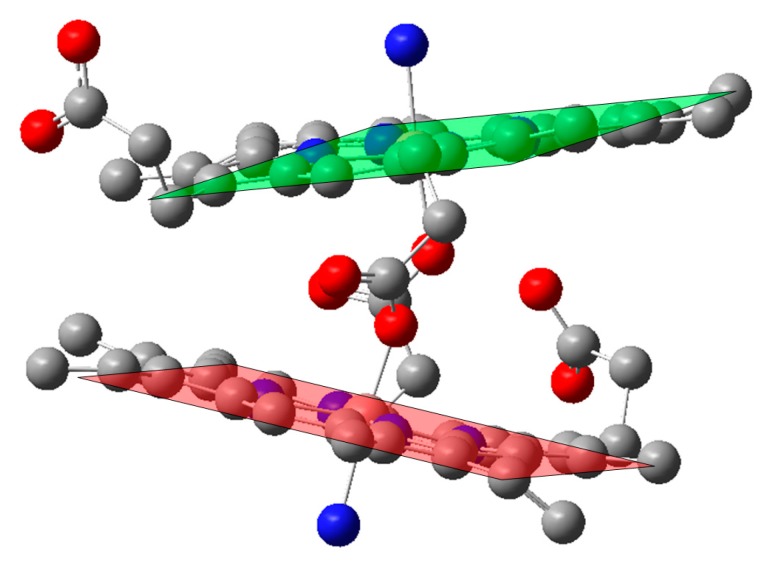
Ball-and-stick representation of the ground state geometry as calculated with DFT at the B3LYP/LANL2DZ level of theory. This DFT-calculated ground state geometry closely resembled the structure obtained later from X-ray analysis [98]. Color coding: grey = carbon, blue = nitrogen, red = oxygen. Reproduced with permission of American Chemical Society [98].

Mack *et al*. [100] demonstrated a series of trends that linked parameters that were extracted from the optical spectra of a wide range of porphyrins and phthalocyanines and correlated these with the energies of the “Gouterman four-orbital set”, *i.e.*, the two highest occupied and the two lowest virtual orbitals, see Figure 11, and the optimized geometry in Figure 12. Those MOs were reproduced by Pinter *et al*. [98] with the data for the Ga(III)-PPIX and Zn(II)-PPIX included.

The key features of the data in this figure are the energy differences between the top two occupied orbitals, which have a_1u_ and a_2u_ symmetry in symmetric porphyrins. The trends above show that as the energy difference between the orbitals increases so does the intensity of the formally-forbidden *Q*-transition. Clearly, the highest occupied MOs of the Ga(III)-PPIX and the Zn(II)-PPIX complexes resemble the splitting of Zn(II)-TPP (tetraphenylporphyrin) rather than Zn(II)-Pc (phthalocyanine).

**Figure 13:**
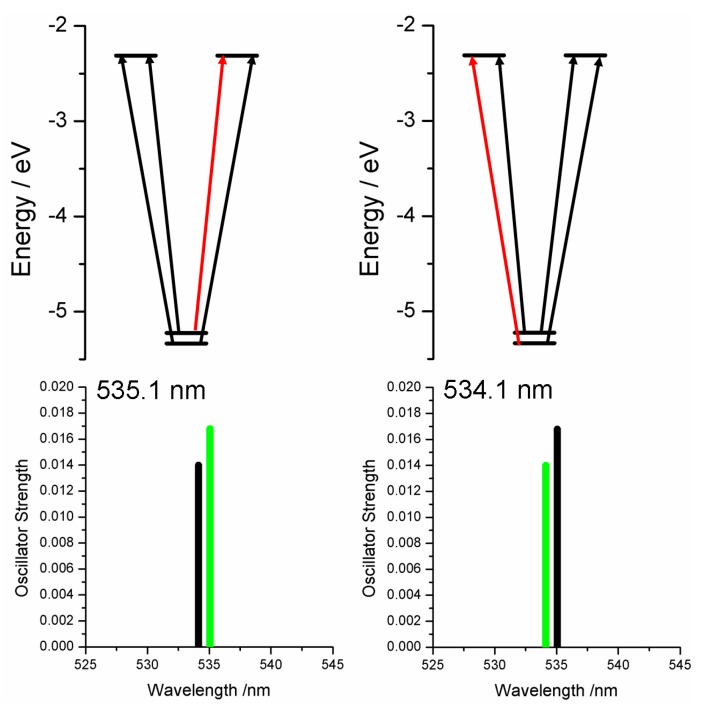
Contributing MOs for the Q-band in Ga(III)-PPIX as obtained from TD-DFT calculations. The Q-band is predicted to be at 535 nm, but is observed at 578 nm. The orbital contributions (black for positive; red for negative) are shown for the green transition at each energy. The black transition is described in the adjacent figures. Two bands are predicted to form the observed Q band, at 535.1 and 534.1 nm. As typically found, the pair of states representing the Q band, result from different combinations of transitions between the same molecular orbitals when the electronic structure of the molecule is not complicated by structural aspects. Reproduced with permission of American Chemical Society [98].

Time-dependent density functional theory (TDDFT) allows predictions of the calculated optical spectrum of molecules. It is our experience that while TDDFT results can reproduce the absorption spectrum over a wide energy range quite well, the accuracy of the predicted Q-band energies are often quite wrong. In particular, we commonly observed blue-shifted optical spectra from TDDFT calculations. On the other hand, while the ZINDO/S spectral calculations often do not reproduce the whole 200–800 nm spectrum well, the calculated Q-band energies actually are usually very good. The Q-band contributions for the Ga(III)-PPIX complex are quite straightforward and reflect on the lack of an interaction of the Ga(III) ion with the π-system. Figure 13 diagrammatically shows the MO contributions to the two overlapping Q-band transitions of the Ga(III)-PPIX complex, which are at almost the same energy as those indicated by the symmetric A-term in Figure 7 above. The Q-band region of the {(Ga(III)-PPIX)_2_} is much more complicated with cross-term contributions that participate significantly in optical data in the Q-band region. The TDDFT calculation predicted a Q-band maximum near 548 nm [98].

The computational and experimental data described here provides strong support for the use of DFT methods to interrogate the observed optical data; however, the error in the energy estimations is a critical problem. The trends described since 2005 and extended here offer a route to circumventing errors in assignments when overlapping bands are present, for example as a result of charge transfer. The value of these trends lies also in their predictive use, calculation of the electronic structure for molecules yet to be synthesized will allow the Q- and B-band energies to be predicted together with the Q-band oscillator strength.

### 2.4. Case Study 4

The two porphyrin rings in the Ga(III)-PPIX dimer exhibit little direct overlap of the orbitals near the HOMO, however, in many dimers there is significant delocalization. It is a goal in the design of molecules that exploit the photophysical and redox properties inherent in the 18 π-system of the porphyrins that they will exhibit very high absorbances in the visible-near infrared (IR) optical region. In addition, red-shifting the Q band transition makes the compound more valuable technologically; for example, in solar cells and for photodynamic therapy. However, an important first question to answer is “will a specific new molecule actually exhibit complete delocalization in the HOMO, or will there be a localized electronic distribution”, as was the case in the Ga(III)-PPIX? A final question could be “what is the predicted color of this new molecule”?

**Figure 14:**
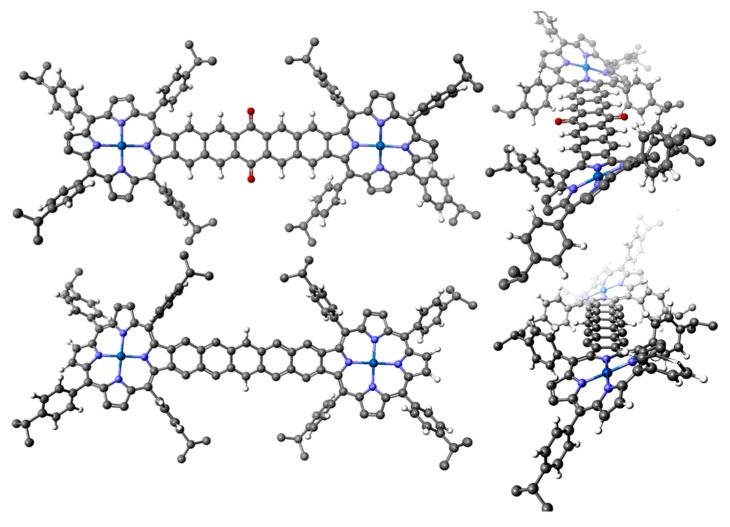
Molecular structures of the two fused-linker Ni-diporphyrins as calculated by density functional theory (DFT) optimization. (**Top**) Cross-conjugated quinone-linked dinaphtho[2,3]Zn-porphyrin (2a); (**Bottom**) Rigid conjugated pentacene peripherally-fused linked di-Ni-porphyrin (1a). Reproduced with permission, Jiang *et al.* [102].

This brings us then to our last example, which is from the work of Hong Wang and colleagues [102] that illustrates the value of the computational approach to understanding the optical properties of very large systems. We mean by this that although the computational results might not provide an exact match of the experimental data (of course, solvent effect and solution broadening are not typically obtained from these TDDFT calculations) the energies and electron density distribution in the molecular orbitals can be used to interpret and analyze the optical data, and, particularly, for porphyrins, the respective MCD spectral data. We would like to use the phrase “massive peripheral substitution” to describe the porphyrins made by Wang because although the molecules shown in Figure 14 are porphyrin dimers, the effect of the conjugated linkers on the MO structure of each of the rings is similar to that of peripheral substitution by a conjugated group. In these dimeric compounds, the linkers between the two porphyrin rings are either a cross-conjugated quinone-dinaphtho- (2a, top) or a rigid conjugated pentacene peripherally-fused ladder (1a, bottom), Figure 14. The key difference between the two structures is the quinone, located midway in the pentacene ladder in 2a; both are substituted by Ni(II). X-ray diffraction analysis confirmed the ruffling of the quinone-based structure that was predicted by the DFT ground state geometry calculations [102].

The ground state geometry minimization carried out using DFT calculations very closely reproduced the structure of the Ni-(quinone-dinaphtho[2,3]diporphyrin) determined by X-ray diffraction (Figure 14; TOP). Both structures show a marked twist across the rigid and almost planar linking ladder of the pentacene-parent. There is only a DFT-optimized geometry structure for the pentacene-Ni-diporphyrin but the DFT optimized structure closely resembles that of the quinone-dinaphtho-Ni-diporphyrin providing support for the calculated structure.

**Figure 15:**
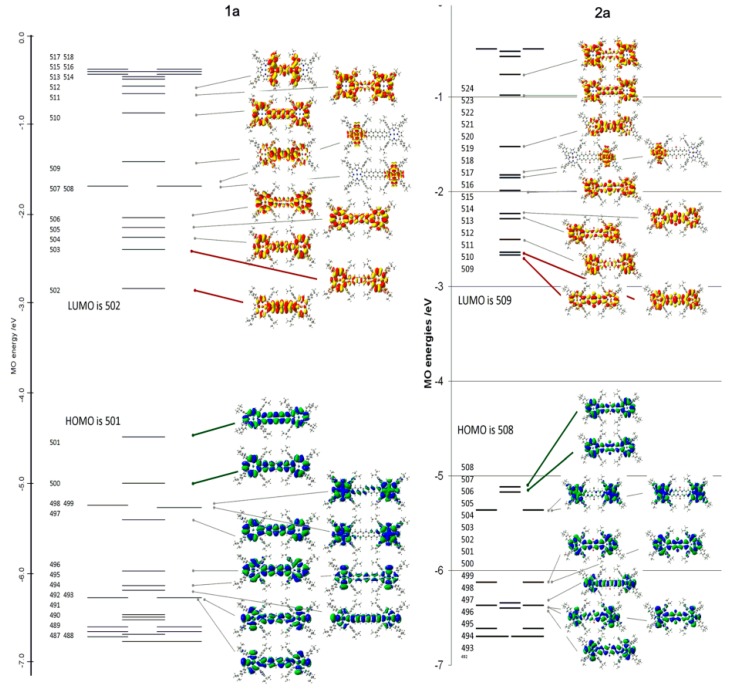
Energies and MO electron density surfaces for 1a and 2a from G09 TDDFT calculations. Of particular note, is the large splitting of the pairs of HOMO and LUMO orbitals in (**1a**) and the almost complete absence of splitting in (**2a**), see Figure 14 for the molecular structures and Figure 16 for a more detailed view with the energies included. Reproduced with permission, Jiang *et al.* [102].

**Figure 16:**
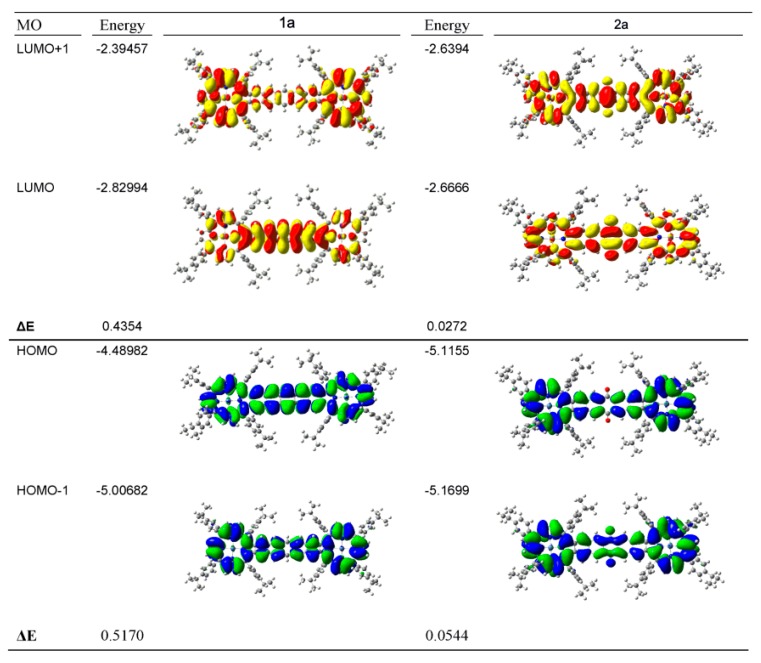
The pairs of HOMO (500/501 (**1a**) and 507/508 (**2a**)) and LUMO (502/503 (**1a**) and 509/510 (**2a**)) orbitals with their respective energies for the pentacene-linked Ni-diporphyrin 1a (**left**) and the quinone-dinaphtho-Ni-diporphyrin 2a (**right**) showing the energy differences (ΔE). Reproduced with permission, Jiang *et al.* [102].

**Figure 17:**
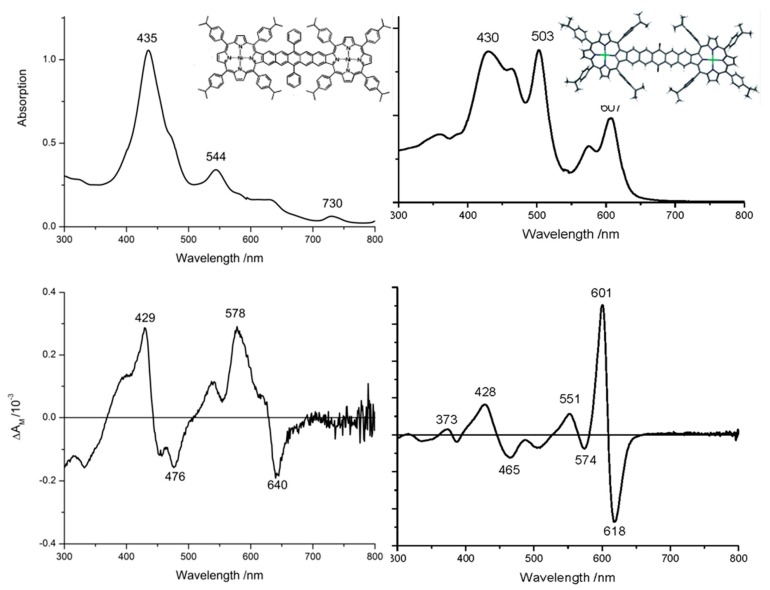
Absorption and MCD of the pentacene-fused Ni-diporphyrin, Compound 1a (**Left**) and the quinone-dinaphtho-Ni-diporphyin, Compound 2a (**Right**). The molecular structures are shown in Figure 14. Adapted from and reproduced with permission, Jiang *et al.* [102].

The MO energy stack (Figure 15 and Figure 16) provide insight into the first and paramount question “Are the highest occupied and lowest unoccupied orbitals delocalized over both porphyrin rings of the diad or localized in the main on just one ring”? A cogent reason for asking this question came from the absorption spectra, Figure 17. The spectrum, Figure 17, of the pentacene-linked-Ni diporphyrin (1a) was unlike a typical porphyrin spectrum. Specifically, the series of bands to the red of 500 nm extending to 750 nm was unusual. Similarly the absorption spectra of the Ni- and Zn- quinone-dinaptho-linked porphyrins (2a and 2b in Ref [102]; just the Ni-2a complex is shown here) were also unusual but not the same as that measured for the Ni-porphyrin with the pentacene linker (1a).

TDDFT calculations were carried out on all four of the compounds reported by Jiang *et al.* [102] Figure 15 shows that clearly for both the Ni-pentacene linker and Ni-quinone-dinaphtho- linker that delocalization is complete especially for the top two filled MOs (for 1a: 501/500; for 2a: 508/507) and the two lowest unoccupied π* orbitals (for 1a: 501/502; for 2a: 509/510). In addition, these MOs are clearly π-ring and pentacene-based. Key data from the energy level Figure 15 is that for the pentacene-linked Ni-porphyrin (1a) highest occupied MOs 500 and 501, and lowest unoccupied 502 and 503 MOs are all well separated in energy, Figure 16. Splitting of the two lowest unoccupied MOs is unusual for a porphyrin.

In Figure 16, the surfaces of the pairs of highest occupied MOs (500/501 (1a) and 507/508 (2a)) and lowest unoccupied MOs (502/503 (1a) and 509/510 (2a)) for the pentacene-linked Ni-diporphyrin 1a (left) and the quinone-dinaphtho-Ni-diporphyrin 2a (right), show the extent of delocalization of these four critical porphyrin MOs. The energies of these four orbitals indicate that the presence of the quinones is to reduce the splitting between the pairs of HOMO and LUMO orbitals compared with the benzenoid pentacene. Surprisingly, the nodal pattern of 4 for the HOMO and 5 for the LUMO expected and found for the monomeric tetrapyrroles is retained in both compounds despite the delocalization across the entire molecule [100]. The order of magnitude difference in the energies between the pairs of occupied and unoccupied orbitals has significant consequences for the angular momentum observed in the MCD spectra as we will discuss next.

The optical data of the two structures were markedly different (Figure 17). Neither compound exhibited either absorption or MCD spectral envelopes similar to typical porphyrin spectra [59] for which typically, one sees a strong B band near 400 nm and a weaker Q band near 580 nm, as we show above for the Zn(II)-PPIX, Figure 8. However, the assignments of the optical data became clearer when the MCD spectra were compared, Figure 17. In the Jiang *et al.* [102] paper, the Ni(II), and free base derivatives of the pentacene-linked diporphyrins (Compound 1a, in Jiang *et al*. [102]) and the Ni-, Zn-, and free-base-quinone-dinaphtho-diporphyrins (Compounds 2a, 2b and 2c in [102]) were reported.

The first answer that the MCD spectra provided was that the Ni(II) derivatives exhibited only one transition in the absorption spectrum not observed for the free-base or Zn(II) derivatives, namely a band at 730 nm in the spectrum of Compound 1a. The other bands in the spectrum could be assigned as being π-π*. The MCD spectral data for Ni-1a are quite different than the data for the Ni- and Zn- quinone-dinaphtho-linked porphyrins. The MO energy diagram (Figure 16) shows how the energies of the top filled and lowest empty MOs are quite different for the 2 classes of linker. For the pentacene linker (Compound 1a), 500 and 501 and 502 and 503 are split by about 0.5 eV. This is significant because it breaks any accidental degeneracies left over from the 18 π rings [100] and as we will explain below, accounts for the MCD spectral envelope for 1a. The split between these four orbitals for the quinone-dinaphtho-Ni and Zn-diporphyrins (Compounds 2a and 2b) is an order of magnitude smaller with 507 and 508 split by *ca.* 0.05 eV and 509 and 510 by just 0.03 eV. Again, it accounts for the observed MCD spectral envelope as we will outline next.

The MCD spectrum of porphyrins has been described above and the assignment power for porphyrinoid spectra outlined. Here in Figure 17 we find the essential assignment criterion provided by the MCD experiment clearly illustrated through the manner in which the change in angular momentum for both pseudo A terms (which are really close lying B terms) and oppositely signed B terms [59] is related to the electronic structure. The MCD spectrum very closely tracks the angular momentum of aromatic compounds, but in the case of the symmetry lowering effect of the fused linkers we were surprised that the porphyrin aromaticity dominated the spectral data for the quinone-dinaphtho linker species.

The effect of the splitting by 0.5 eV of MOs 500/501 and 502/503 is to separate the nominally degenerate pairs of porphyrin ring MOs. The MCD clearly shows the presence of a series of coupled B terms located under each of the many bands in the absorption spectrum of Compound 1a. The Ni-pentacene-linked-diporphyrin spectrum could be assigned to a series of nondegenerate transitions from the occupied orbitals 497–501 to 502–506. Thus, we can identify bands in the porphyrin B region near the maximum at 435 nm with MCD bands at 429 (+), and 476 (−) nm as representing most likely superposition of four transitions, then the 544 nm absorption is associated with a weak positive MCD band followed by coupled MCD B terms at 578 and 640 nm. This accounts for all but the 730 nm band. The MCD is very weak (and noisy) but might arise from charge transfer between the ring and the Ni(II).

For the same approximate absorbance in the B region the Ni-quinone-linked-diporphyrin spectrum is also assigned to a series of nondegenerate transitions but the top filled (507/508) and lowest unoccupied (509/510) are so close in energy that we now observe a very much more intense set of bands as the angular momentum has not been quenched as much. So, the MCD spectrum clearly identifies the transitions at 428 (+) and 465 (−) nm, the 503 nm band appears associated with the negative MCD band at 500 nm. It is the visible region bands that illustrate best the assignment power of the technique. The effect of the near degeneracy of the 507/508 and 509/510 MOs is signally by the much more intense pseudo A term at 601(+) and 618(−) nm.

In summary for Case 4, we find that the combination of the MCD data and the calculation of the energies and special distribution of the molecular orbitals together allowed characterization of the optical properties of these unusual molecules.

## 3. Conclusions

Computational modelling carried out for metalloporphyrins and hemes is an important branch of research and provides a strong foundation for experimental studies into structure, mechanism and reactivity. Often computational modelling can give insights beyond those that are found from experiment and frequently helps with the interpretation of spectroscopic results. This present paper highlights a series of case studies describing metalloporphyrins focused on understanding the detailed structure, reactivities, spectroscopic parameters and mechanisms leading to an understanding of the contribution of each molecular component to the observed reactivity, redox properties and optical properties, and ultimately allowing informed design of novel compounds for technological and medical application.

In Case Study 1, we emphasize reproducing experimentally observed reaction mechanisms, product distributions and rate constants. As iron-based complexes tend to have close-lying spin states this is not a straightforward task. Nevertheless, computational modelling reproduces the experiment well and gives free energies of activation that follow the experimental trends. Moreover, despite an apparent systematic error, theory predicts the correct regio- and enantioselectivity of chemical reactions.

In Case Study 2, work on biomimetic model complexes is described, which in contrast to enzyme chemistry proceed in the solvent phase rather than in an encapsulated protein structure. The described work predicts rate enhancements and kinetic isotope effects well and generally explains experimental observations.

In Case Study 3, the spectroscopic properties of metalloporphyrins are described. In particular focus is in Ga(III) and Zn(II) porphyrins, models for the heme. It is shown that a variety of spectroscopic features are obtained through small modifications to the porphyrin structure and substituents, which is described in terms of orbital energy levels and excitation energies.

In Case Study 4, we focus on understanding and predicting the optical properties of extremely complicated porphyrin structures, here porphyrins linked by aromatic “ladders”. Computational analysis has relevance to the prediction of the color of a molecule. The examples show that the calculations generally do well and predict the correct spectroscopic features.

## Figures and Tables

**Table 1:** HOMO/LUMO energy gaps in Ga(III) and Zn(II)-PPIX complexes. Values are in kcal·mol^−1^.

Compound ^1^	Δ{HOMO − (HOMO − 1)}	Δ{(LUMO + 1) − (LUMO)}	Δ{LUMO − HOMO}
Ga(III)-PPIX	0.109	~0	23.20
Zn(II)-PPIX	0.054	~0	23.70

^1^ Data adapted from [91].
